# Effect of Sacubitril/Valsartan, Ivabradine, and Captopril on Anxiety-like Behavior in Spontaneously Hypertensive Rats

**DOI:** 10.3390/ijms262210905

**Published:** 2025-11-10

**Authors:** Maria Szighardtova, Silvia Aziriova, Peter Stanko, Kristina Repova, Tomas Baka, Kristina Krajcirovicova, Stefan Zorad, Michaela Adamcova, Peter Sabaka, Veronika Borbélyová, Fedor Simko

**Affiliations:** 1 Institute of Pathophysiology, Faculty of Medicine, Comenius University, 811 08 Bratislava, Slovakiasilvia.aziriova@fmed.uniba.sk (S.A.); peter.stanko23@fmed.uniba.sk (P.S.); repova.k@gmail.com (K.R.); tomasko.baka@gmail.com (T.B.); krikratina@gmail.com (K.K.); 2 Institute of Experimental Endocrinology, Biomedical Research Center, Slovak Academy of Sciences, 845 05 Bratislava, Slovakia; stefan.zorad@savba.sk; 3 Department of Physiology, Faculty of Medicine, Charles University, 500 03 Hradec Kralove, Czech Republic; adamcova@lfhk.cuni.cz; 4 Department of Infectiology and Geographical Medicine, Faculty of Medicine, Comenius University, 813 72 Bratislava, Slovakia; petersabaka@gmail.com; 5 Institute of Molecular Biomedicine, Faculty of Medicine, Comenius University, 811 08 Bratislava, Slovakia; veronika.borbelyova@fmed.uniba.sk; 6 3rd Department of Internal Medicine, Faculty of Medicine, Comenius University, 833 05 Bratislava, Slovakia

**Keywords:** spontaneously hypertensive rats, anxiety, sacubitril/valsartan, captopril, ivabradine, blood pressure, heart rate, correlation analysis

## Abstract

Cardiovascular disorders and the medications used to treat them can affect physiological patterns of behavior. The aim of the present study was to determine whether the dual inhibition of neprilysin and angiotensin II—sacubitril/valsartan (ARNI) can modify anxiety-like behavior in male spontaneously hypertensive rats (SHR). We compared ARNI with two other drugs in the portfolio of heart failure treatment, captopril and ivabradine. Six groups (n = 13) of 12-week-old rats were treated for six weeks: control (Wistar rats), control + ARNI, SHR, SHR + ARNI, SHR + captopril, and SHR + ivabradine. The elevated plus maze test, the open field test, and the light–dark box test were used to determine anxiety-like behavior. SHRs exhibited higher systolic blood pressure (SBP), heart rate (HR), left ventricular weight (LVW), and hydroxyproline concentration (LVHP) but displayed a reduced level of anxiety-like behavior in comparison to controls. ARNI reduced SBP, HR, and LVW but had no significant effect on the level of anxiety in SHR, and similar results were achieved by captopril and ivabradine. Additionally, correlation analysis indicated that anxiety-like behavior in Wistar rats or SHR, either with or without cardiovascular therapy, was independent of SBP, HR, LVW, or LVHP. The level of anxiety-like behavior can, therefore, be considered part of the inherent neurobehavioral traits unrelated to fundamental hemodynamic or structural cardiovascular parameters.

## 1. Introduction

Serious pathological conditions, including cardiovascular diseases (CVDs), can lead to changes in behavior in the form of anxiety and depression. On the other hand, the persistence of anxious and depressive moods tends to be reflected in an increased incidence of CVDs, such as coronary artery disease, myocardial infarction, type 2 diabetes, hypertension, or heart rate disturbances [1,2,3,4,5]. In addition to the pathological conditions themselves, pharmacological or non-pharmacological interventions targeted at cardiovascular pathologies can also modify physiological behavioral patterns [6,7,8]. It is, therefore, of the utmost importance to assess the potential beneficial or adverse effects of pharmacological agents on behavioral manifestations, as this could facilitate a more comprehensive and targeted approach to cardiovascular interventions [9].

Severe cardiovascular pathologies are associated with the activation of neurohumoral systems, such as the renin–angiotensin–aldosterone system (RAAS) and the adrenergic system [10,11]. Angiotensin II (Ang II), aldosterone, and catecholamines can contribute to psychological alterations related to the onset and progression of anxiety. On the other hand, RAAS inhibitors and sympathetic system blockers may exert anxiolytic effects [9,12,13,14,15,16]. The dual inhibitor of neprilysin and Ang II sacubitril/valsartan (ARNI) has been introduced relatively recently in the treatment of heart failure. Sacubitril inhibits the endopeptidase neprilysin, which degrades several peptides, including atrial and brain natriuretic peptides (ANP/BNP), as well as Ang II. By blocking this enzyme, sacubitril supposedly increases ANP/BNP levels, whose vasodilatory, diuretic, and antiproliferative effects are considered beneficial to cardiac function. The association of sacubitril with the Ang II type 1 receptor (AT1R) blocker valsartan exploits both the antiproliferative and vasodilative effects of natriuretic peptides while preventing the undesirable proliferative action of Ang II [17]. The complex compound ARNI, a dual inhibitor of AT1R for Ang II and neprilysin, reduced cardiovascular events in patients with moderate systolic heart failure [18,19].

There is evidence in the literature suggesting that inhibition of the formation or effects of Ang II by angiotensin-converting enzyme (ACE) inhibitors or AT1R blockers has anxiolytic effects [20,21,22]. In addition to blocking the effects of Ang II by valsartan, ARNI not only inhibits the cleavage of ANP/BNP through sacubitril-induced neprilysin inhibition, but also limits the degradation of enkephalins, substance P, vasoactive intestinal peptide, or bradykinin, the levels of which are increased [17]. While ANP [23], enkephalins [24], and substance P [25] have shown anxiolytic effects, bradykinin has a rather anxiogenic effect in rats [26]; therefore, the effect of ARNI on anxiety is complex and difficult to predict. Moreover, although there are isolated data on the anxiolytic effect of ARNI in patients with heart failure [27,28,29], data for ARNI on anxiety behavior in hypertension are completely lacking and remain a research challenge. Thus, the aim of the current study was to determine whether ARNI can modify anxiety-like behavior in SHRs. We compared ARNI with two other drugs used in heart failure therapy: the classic ACE inhibitor captopril and the hyperpolarization-activated cyclic nucleotide-gated ‘funny’ channel (If) blocker ivabradine, which exerts a bradycardic effect. Furthermore, we aimed to determine whether there was any relationship between behavioral changes in untreated or treated SHRs and Wistar rats and basic cardiovascular hemodynamic and structural parameters.

## 2. Results

### 2.1. Analysis of Hemodynamic Parameters and Cardiac Structural Parameters

Systolic blood pressure (SBP). Treatment had a significant effect on SBP during the 6-week treatment period (*F*(5,72) = 107.05, *p* < 0.001) as well as after its conclusion (*F*(5,72) = 44.28, *p* < 0.001).

**Figure 1:**
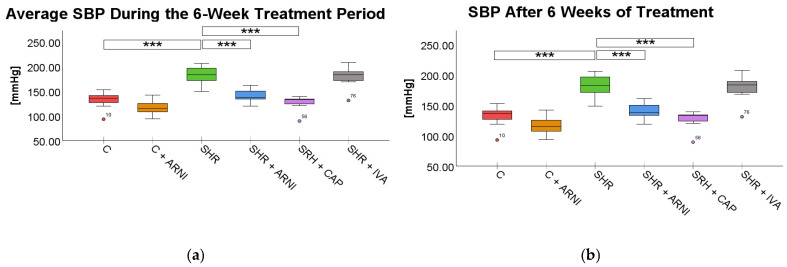
Effects of ARNI, captopril, and ivabradine on (**a**) average systolic blood pressure (SBP) during the 6-week treatment period and (**b**) SBP after 6 weeks of treatment in Wistar control rats (C) and spontaneously hypertensive rats (SHRs). Data are presented as means ± SD. One-way ANOVA was performed, followed by the Bonferroni post hoc test; *** Significance at *p* < 0.001. Abbreviations: ARNI—sacubitril/valsartan; CAP—captopril; and IVA—ivabradine.

The average SBP during the treatment period was elevated in SHRs (184.43 ± 2.51 mmHg) compared to the Wistar control (C) group (128.65 ± 2.39 mmHg; *t*(72) = 16.12, *p* < 0.001). Treatment with ARNI (154.56 ± 1.75 mmHg vs. SHR; *t*(72) = 8.64, *p* < 0.001) and captopril (142.54 ± 1.89 mmHg; *t*(72) = 12.11, *p* < 0.001) significantly reduced SBP in SHR. In contrast, ivabradine treatment in SHRs did not lead to a statistically significant reduction in average SBP (176.97 ± 3.69 mmHg vs. SHR; *t*(72) = 2.16, *p* = 0.51; Figure 1a).

At the end of the treatment period, SBP remained significantly elevated in SHRs (182.50 ± 4.78 mmHg) compared to the C group (131.94 ± 4.20 mmHg; *t*(72) = 8.54, *p* < 0.001). ARNI treatment again significantly reduced SBP (141.12 ± 3.40 mmHg vs. SHR; *t*(72) = 6.99, *p* < 0.001), with the most pronounced reduction observed in the captopril-treated SHRs (128.00 ± 3.61 mmHg vs. SHR; *t*(72) = 9.20, *p* < 0.001). Ivabradine did not produce a statistically significant reduction at the end of the treatment period (179.41 ± 5.08 mmHg vs. SHR; *t*(72) = 0.52, *p* = 0.99; Figure 1b).

Heart rate (HR). HR was also significantly influenced by treatment both during the 6-week treatment period (*F*(5,72) = 30.33, *p* < 0.001) and after 6 weeks of treatment (*F*(5,72) = 11.02, *p* < 0.001).

**Figure 2:**
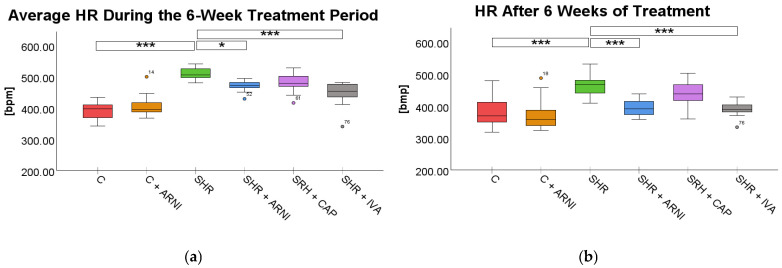
Effects of ARNI, captopril, and ivabradine on (**a**) average heart rate (HR) during the 6-week treatment period and (**b**) HR after 6 weeks of treatment in Wistar control rats (C) and spontaneously hypertensive rats (SHRs). Data are presented as means ± SD. One-way ANOVA was performed, followed by the Bonferroni post hoc test; * Significance at *p* < 0.05; *** Significance at *p* < 0.001. Abbreviations: ARNI—sacubitril/valsartan; CAP—captopril; and IVA—ivabradine.

The average HR during treatment was significantly higher in SHRs (508.71 ± 5.49 bpm) compared to the C group (392.27 ± 7.95 bpm; *t*(72) = 10.15, *p* < 0.001). Treatment with ARNI (471.58 ± 4.69 bpm; *t*(72) = 3.24, *p* = 0.03) and ivabradine (446.56 ± 10.57 bpm; *t*(72) = 5.42, *p* < 0.001) significantly reduced HR in SHR. In contrast, captopril treatment did not result in a statistically significant reduction in average HR (480.27 ± 8.62 bpm vs. SHR; *t*(72) = 2.48, *p* = 0.23; Figure 2a).

After 6 weeks of treatment, HR remained significantly elevated in SHRs (468.73 ± 10.99 bpm) in comparison to the C group (379.85 ± 12.88 bpm; *t*(72) = 5.64, *p* < 0.001). Both ARNI (394.43 ± 7.84 bpm vs. SHR; *t*(72) = 4.71, *p* < 0.001) and ivabradine (392.86 ± 7.00 bpm vs. SHR; *t*(72) = 4.81, *p* < 0.001) significantly reduced HR in SHRs at the endpoint. However, captopril treatment (436.41 ± 12.71 bpm) did not produce a significant difference compared to untreated SHRs (*t*(72) = 2.05, *p* = 0.66; Figure 2b).

**Figure 3:**
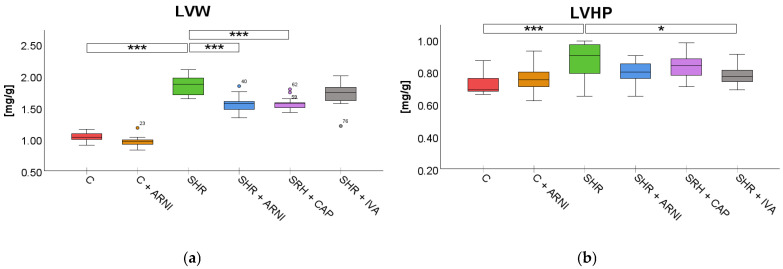
Effects of ARNI, captopril, and ivabradine on (**a**) left ventricular weight (LVW) and (**b**) left ventricular hydroxyproline concentration (LVHP) in Wistar control rats (C) and spontaneously hypertensive rats (SHRs) after six weeks of treatment. Data are presented as means ± SD. One-way ANOVA was performed, followed by the Bonferroni post hoc test; * Significance at *p* < 0.05; *** Significance at *p* < 0.001. Abbreviations: ARNI—sacubitril/valsartan; CAP—captopril; and IVA—ivabradine.

Left ventricular weight (LVW). LVW was significantly affected by treatment (*F*(5,72) = 95.23, *p* < 0.001). SHRs exhibited a substantial increase in LVW (1.85 ± 0.04 mg/g) compared to the C group (1.04 ± 0.02 mg/g; *t*(72) = 16.20, *p* < 0.001). This increase was significantly attenuated by treatment with ARNI (1.56 ± 0.04 mg/g vs. SHR; *t*(72) = 5.80, *p* < 0.001) and captopril (1.57 ± 0.03 mg/g vs. SHR; *t*(72) = 5.60, *p* < 0.001) in SHR. In contrast, ivabradine did not significantly reduce the LVW in SHRs (1.71 ± 0.06 mg/g vs. SHR; *t*(72) = 2.80, *p* = 0.14; Figure 3a).

Left ventricular hydroxyproline concentration (LVHP). Treatment also significantly influenced LVHP (*F*(5,72) = 5.22, *p* < 0.001). SHRs had elevated LVHP levels (0.87 ± 0.03 mg/g) compared to the C group (0.74 ± 0.02 mg/g; *t*(72) = 4.67, *p* < 0.001). Among treatments in SHR, only ivabradine significantly reduced the concentration of LVHP (0.78 ± 0.02 mg/g vs. SHR; *t*(72) = 3.34, *p* = 0.047). ARNI (0.80 ± 0.02 mg/g vs. SHR; *t*(72) = 2.34, *p* = 0.41) and captopril (0.84 ± 0.02 mg/g vs. SHR; *t*(72) = 1.34, *p* = 0.99) did not produce statistically significant changes in the LVHP of SHRs (Figure 3b).

### 2.2. Anxiety-like Behavior

**Figure 4:**
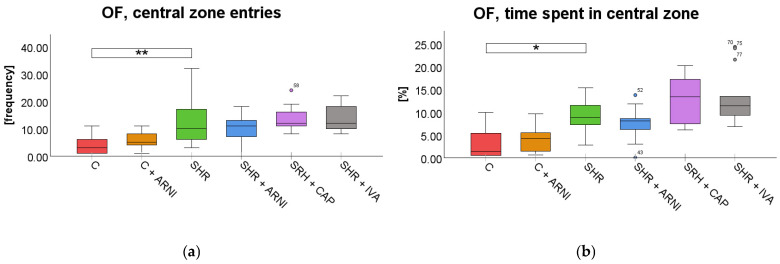
Effect of ARNI, captopril, and ivabradine on anxiety-like behavior in Wistar control rats (C) and spontaneously hypertensive rats (SHRs) in the open field test (OF): (**a**) Frequency of central zone entries in the OF; and (**b**) percentage of time spent in the central zone in the OF. Data are presented as means ± SD. One-way ANOVA was performed, followed by the Bonferroni post hoc test; * Significance at *p* < 0.05; ** Significance at *p* < 0.01. Abbreviations: ARNI—sacubitril/valsartan; IVA—ivabradine; and CAP—captopril.

Open Field Test (OF). In the OF test, a significant overall difference in the frequency of entries into the central zone was found among the groups (*F*(5,72) = 8.41, *p* < 0.001). SHRs exhibited significantly more entries into the central zone (12 ± 2) compared to the C group (4 ± 1; *t*(72) = 4.01, *p* = 0.002). However, treatment with ARNI (10 ± 1 vs. SHR; *t*(72) = 0.83, *p* = 0.99), captopril (13 ± 1 vs. SHR; *t*(72) = 0.65, *p* = 0.99), or ivabradine (14 ± 1 vs. SHR; *t*(72) = 0.72, *p* = 0.99) did not significantly change the frequency of central zone entries in SHRs (Figure 4a).

Similarly, a significant overall difference in time spent in the central zone in the OF was found among the groups (*F*(5,72) = 13.32, *p* < 0.001). SHRs spent significantly more time in the central zone (8.72 ± 1.02%) compared to the C group (3.10 ± 0.92%; *t*(72) = 3.41, *p* = 0.02). However, this increase in SHRs was not significantly modified by treatment with ARNI (7.23 ± 1.00% vs. SHR; *t*(72) = 0.90, *p* = 0.99), captopril (12.58 ± 1.39% vs. SHR; *t*(72) = 2.35, *p* = 0.33), or ivabradine (13.18 ± 1.68% vs. SHR; *t*(72) = 2.71, *p* = 0.13; Figure 4b).

**Figure 5:**
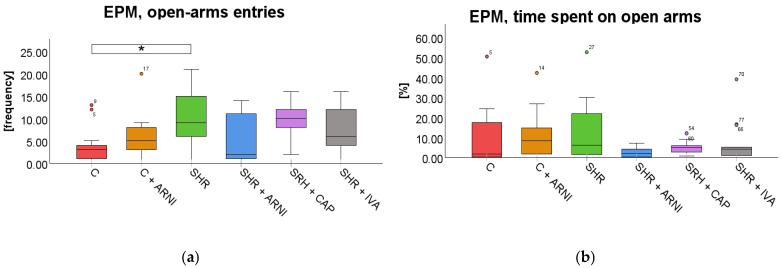
Effect of ARNI, captopril, and ivabradine on anxiety-like behavior in Wistar control rats (C) and spontaneously hypertensive rats (SHRs) in the elevated plus maze test (EPM): (**a**) Frequency of open-arm entries in the EPM; (**b**) and percentage of time spent on open arms in the EPM. Data are presented as means ± SD. One-way ANOVA was performed, followed by the Bonferroni post hoc test; * Significance at *p* < 0.05. Abbreviations: ARNI—sacubitril/valsartan; IVA—ivabradine; and CAP—captopril.

Elevated Plus Maze Test (EPM). In the EPM test, a significant overall difference in the frequency of entries into the open arms was found among the groups (*F*(5,72) = 2.97, *p* = 0.02; Figure 5a), whereas the time spent on open arms did not differ significantly across groups (*F*(5,72) = 1.93, *p* = 0.10; Figure 5b). SHRs exhibited significantly more entries into the open arm (10 ± 2) than the C group (4 ± 1; *t*(72) = 3.17, *p* = 0.03). However, treatment with ARNI (5 ± 2; *t*(72) = 2.45, *p* = 0.25), captopril (9± 1; *t*(72) = 0.45, *p* = 0.99), and ivabradine (8 ± 1; *t*(72) = 1.32, *p* = 0.99) did not significantly affect the frequency of entries on the open arm compared to SHR.

**Figure 6:**
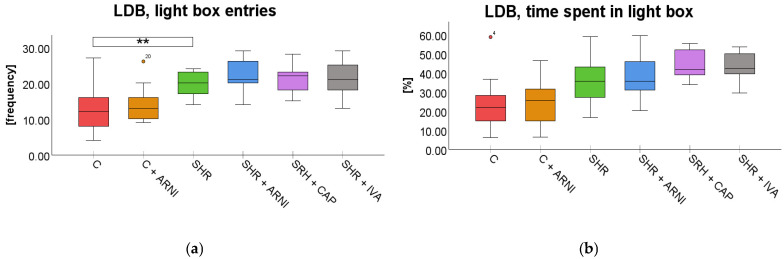
Effect of ARNI, captopril, and ivabradine on anxiety-like behavior in Wistar control rats (C) and spontaneously hypertensive rats (SHRs) in the light–dark box test (LDB): (**a**) Frequency of light box entries in the LDB; and (**b**) percentage of time spent in the LDB light zone. Data are presented as means ± SD. One-way ANOVA was performed, followed by the Bonferroni post hoc test; ** Significance at *p* < 0.01. Abbreviations: ARNI—sacubitril/valsartan; IVA—ivabradine; and CAP—captopril.

Light–Dark Box Test (LDB). In the LDB test, a significant overall difference in the frequency of entries into the light box was found among the groups (*F*(5,72) = 8.87, *p* < 0.001). SHR entered the light box significantly more frequently (20 ± 1) than the C group (13 ± 2; *t*(72) = 3.85, *p* = 0.004). However, this increased frequency in SHRs was not significantly altered by treatment with ARNI (22 ± 1 vs. SHR + ARNI; *t*(72) = 0.97, *p* = 0.99), captopril (21 ± 1 vs. SHR + CAP; *t*(72) = 0.36, *p* = 0.99), or ivabradine (21 ± 1 vs. SHR + IVA; *t*(72) = 0.48, *p* = 0.99; Figure 6a).

A significant overall difference in the time spent in the light compartment in the LDB was found among the groups (*F*(5,72) = 8.12, *p* < 0.001). Despite the overall effect, post hoc analysis showed that SHRs (35.58 ± 3.64%; *t*(72) = 2.74, *p* = 0.12) did not differ significantly from the C group (23.38 ± 3.68%). Similarly, administration of ARNI (37.86 ± 3.26%; *t*(72) = 0.51, *p* = 0.99), captopril (44.13 ± 2.02%; *t*(72) = 1.92, *p* = 0.88), or ivabradine (43.78 ± 2.10%; *t*(72) = 1.84, *p* = 0.99) did not result in statistically significant changes compared to SHRs (Figure 6b).

### 2.3. Association Between Cardiovascular and Anxiety-like Behavior

To explore the potential relationship between cardiovascular parameters and anxiety-like behavior, correlation analyses were performed. The following cardiovascular variables were included in the analysis: SBP, HR, LVW, and LVHP. These parameters were evaluated in relation to behavioral outcomes of the OF, EPM, and LDB tests.

#### 2.3.1. Correlation Analysis in Untreated and ARNI-Treated Control Groups

**Figure 7:**
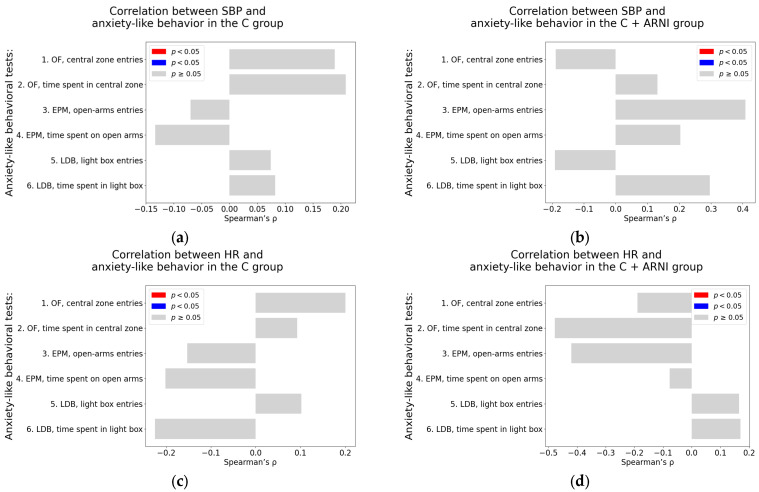

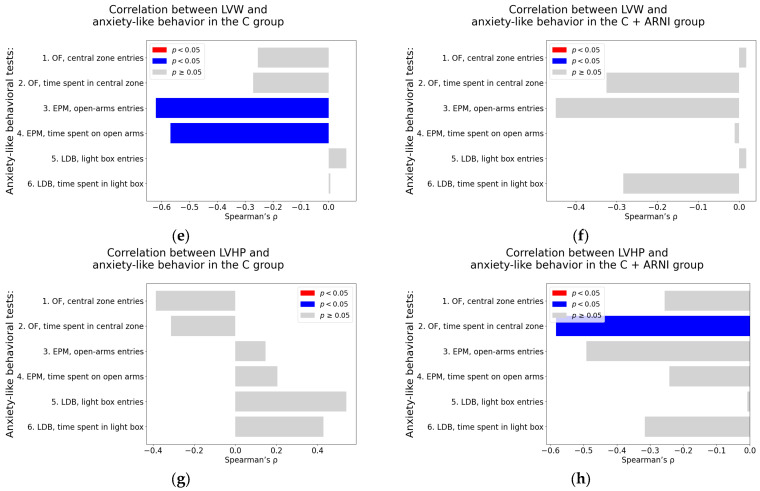
Associations between cardiovascular parameters and anxiety-like behavior in untreated and ARNI-treated Wistar rats: (**a**–**h**) Associations between cardiovascular parameters and anxiety-like behavior; (**a**,**b**) systolic blood pressure (SBP); (**c**,**d**) heart rate (HR); (**e**,**f**) left ventricular weight (LVW); and (**g**,**h**) left ventricular hydroxyproline concentration, each analyzed in untreated controls (C) and ARNI-treated controls (C + ARNI), respectively. Anxiety-like behavior was assessed using the open field test (OF), the elevated plus maze test (EPM), and the light–dark box test (LDB). Nonparametric correlation analysis was used to evaluate the associations; Spearman’s rho with the corresponding *p*-value is reported. Color coding: red bars indicate Spearman’s rho > 0 with *p* < 0.05; blue bars indicate Spearman’s rho < 0 with *p* < 0.05; and gray bars represent no significant association. Abbreviations: C—Wistar controls; and ARNI—sacubitril/valsartan.

Systolic blood pressure. Both in the untreated C group and the C + ARNI group, no significant correlations were observed between SBP and any of the anxiety-like behavioral parameters assessed in the OF, EPM, or LDB tests. All associations were non-significant (*p* > 0.05), as detailed in Table A1 and Table A2 (see also Figure 7a,b).

Heart rate. Similarly, HR was not significantly associated with any of the anxiety-like behavioral measures in either the C group or the C + ARNI group (Table A1 and Table A2; Figure 7c,d).

Left ventricular weight. In the untreated C group, most behavioral parameters did not show significant correlation with LVW. However, significant negative correlations were identified in the EPM for the number of open-arm entries (Spearman’s rho = −0.62, *p* = 0.02) and the time spent on the open arms in the EPM (Spearman’s rho = −0.57, *p* = 0.04) (Table A1; Figure 7e). No significant correlation between LVW and anxiety-like behavior parameters was observed in the C + ARNI group (Table A2; Figure 7f).

Left ventricular hydroxyproline concentration. In the C group, a borderline positive association was found between LVHP and the number of entries into the light box of the LDB (Spearman’s rho = 0.54, *p* = 0.06) (Table A1; Figure 7g). In contrast, the C + ARNI group exhibited a significant negative correlation between LVHP and the time spent in the central zone in the OF (Spearman’s rho = −0.58, *p* = 0.04) (Table A2; Figure 7h).

#### 2.3.2. Correlation Analysis in Untreated and ARNI-Treated Spontaneously Hypertensive Rats

**Figure 8:**
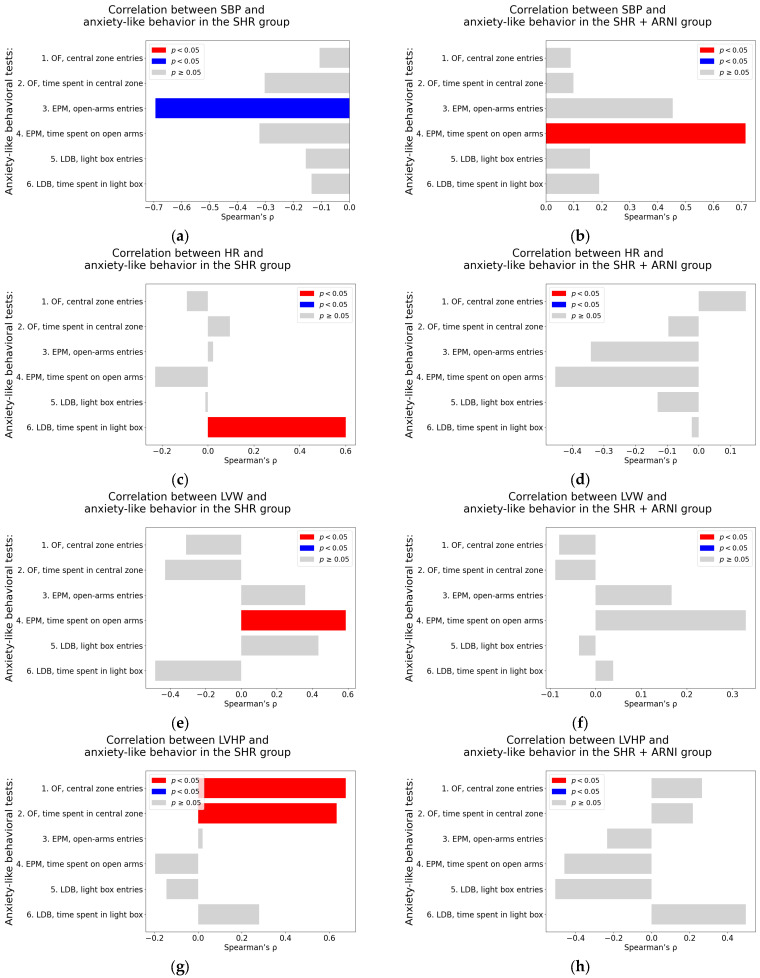
Associations between cardiovascular parameters and anxiety-like behavior in untreated and ARNI-treated spontaneously hypertensive rats: (**a**–**h**) Associations between cardiovascular parameters and anxiety-like behavior; (**a**,**b**) systolic blood pressure (SBP); (**c**,**d**) heart rate (HR); (**e**,**f**) left ventricular weight (LVW); and (**g**,**h**) left ventricular hydroxyproline concentration, each analyzed in untreated spontaneously hypertensive rats (SHR) and ARNI-treated SHRs (SHR + ARNI), respectively. Anxiety-like behavior was assessed using the open field test (OF), the elevated plus maze test (EPM), and the light–dark box test (LDB). Nonparametric correlation analysis was used to evaluate the associations; Spearman’s rho with the corresponding *p*-value is reported. Color coding: red bars indicate Spearman’s rho > 0 with *p* < 0.05; blue bars indicate Spearman’s rho < 0 with *p* < 0.05; and gray bars represent no significant association. Abbreviations: ARNI—sacubitril/valsartan.

Systolic blood pressure. In untreated SHR, a significant negative correlation was observed between SBP and the number of open-arm entries in the EPM (Spearman’s rho = −0.69, *p* = 0.009) (Table A3; Figure 8a). In contrast, the SHR + ARNI group exhibited a significant positive association between SBP and the time spent on an open arm in the EPM (Spearman’s rho = 0.71, *p* = 0.006; Table A4; Figure 8b).

Heart rate. In the untreated SHR group, HR showed a significant positive correlation with the time spent in the light box in the LDB (Spearman’s rho = 0.60, *p* = 0.03; Table A3; Figure 8c). However, in the SHR + ARNI group, no significant correlations were found between HR and any of the anxiety-like behavioral parameters (Table A4; Figure 8d).

Left ventricular weight. In the untreated SHR group, LVW was significantly correlated positively with the time spent on an open arm in the EPM (Spearman’s rho = 0.59, *p* = 0.03; Table A3; Figure 8e). In the SHR + ARNI group, no significant correlations were observed between LVW and anxiety-related behaviors (Table A4; Figure 8f).

Left ventricular hydroxyproline concentration. In the untreated SHR, LVHP was significantly associated positively with both the number of entries into the central zone in the OF (Spearman’s rho = 0.68, *p* = 0.01) and the time spent in the central zone in the OF (Spearman’s rho = 0.63, *p* = 0.02; Table A3; Figure 8g). No significant correlations between LVHP and behavioral parameters were detected in the SHR + ARNI group (Table A4; Figure 8h).

#### 2.3.3. Correlation Analysis in Captopril and Ivabradine-Treated Spontaneously Hypertensive Rats

**Figure 9:**
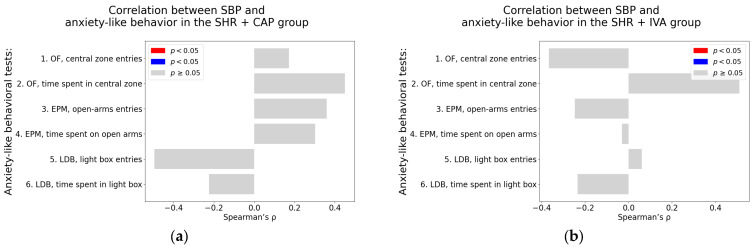

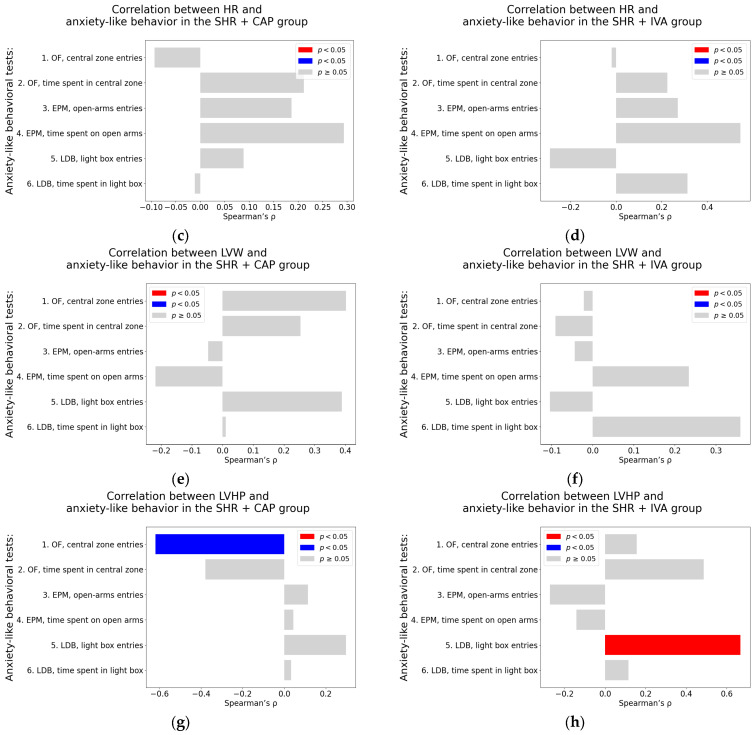
Associations between cardiovascular parameters and anxiety-like behavior in captopril and ivabradine-treated spontaneously hypertensive rats (SHRs); (**a**–**h**) associations between cardiovascular parameters and anxiety-like behavior; (**a**,**b**) systolic blood pressure (SBP); (**c**,**d**) heart rate (HR); (**e**,**f**) left ventricular weight (LVW); (**g**,**h**) left ventricular hydroxyproline concentration, each analyzed in ivabradine-treated spontaneously hypertensive rats (SHR + IVA) and captopril-treated SHRs (SHR + CAP), respectively. Anxiety-like behavior was assessed using the open field test (OF), the elevated plus maze test (EPM), and the light–dark box test (LDB). Nonparametric correlation analysis was used to evaluate the associations; Spearman’s rho with the corresponding *p*-value is reported. Color coding: red bars indicate Spearman’s rho > 0 with *p* < 0.05; blue bars indicate Spearman’s rho < 0 with *p* < 0.05; and gray bars represent no significant association.

Systolic blood pressure. In the SHR + CAP group, SBP did not show any significant correlation with anxiety-like behavioral parameters (Table A5; Figure 9a). In the SHR + IVA group, a borderline positive association was observed between SBP and the time spent in the central zone of the OF (Spearman’s rho = 0.51, *p* = 0.07) (Table A6; Figure 9b).

Heart rate. No significant correlations were found between HR and behavioral parameters in the SHR + CAP group (Table A5; Figure 9c). In the SHR + IVA group, a borderline positive association was detected between HR and the time spent on the open arm in the EPM (Spearman’s rho = 0.55, *p* = 0.05; Table A5; Figure 9d).

Left ventricular weight. Neither the SHR + CAP nor the SHR + IVA group exhibited significant correlations between LVW and any of the anxiety-like behavioral parameters. All associations were non-significant (*p* > 0.05), as detailed in Table A5 and Table A6 (see also Figure 9e,f).

Left ventricular hydroxyproline concentration. In the SHR + CAP group, a significant negative correlation was observed between LVHP and the number of entries into the central zone in the OF (Spearman’s rho = −0.62, *p* = 0.02; Table A5, Figure 9g). In contrast, in the SHR + IVA group, LVHP was significantly associated positively with the number of entries into the light box in the LDB (Spearman’s rho = 0.67, *p* = 0.01; Table A5; Figure 9h).

## 3. Discussion

### 3.1. Observation of Lower Anxiety-like Behavior in SHR

The co-occurrence of anxiety and hypertension is a well-recognized phenomenon in clinical practice and is associated with a poor prognosis [30]. Identifying indicators and elucidating mechanisms underlying a potential causal relationship between hypertension and anxiety, as well as exploring therapeutic interventions that may target both cardiovascular and psychological alterations, remains an important and intriguing area of research. In this study, the observation of lower anxiety-like behavior in SHRs compared to Wistar control rats is partially consistent with previous results from our laboratory, when SHRs spent more time in, and showed higher frequency of entries into the central zone of the OF compared to Wistar control rats [6]. Similarly, SHRs were more active in the OF [31]; they crossed the inside–outside line more often and exhibited more time inside the square than Wistar-Kyoto rats (WKY) [32], indicating lower anxiety-like behavior [31,32]. Also, other laboratories showed similar findings when SHRs were tested by integrating OF, EPM, and LDB tests; they exerted less anxiety-like behavior compared to Lewis rats [33], and SHRs exhibited less anxiety in conflict tests compared to Wistar of WKY rats [34]. In line with this, social isolation did not induce increased cortisol release or an elevated level of depression or anxiety-like behavior in SHRs [35], and maternal separation failed to produce anxiety-like behavior in SHRs [36], suggesting that SHRs are relatively resilient to chronic isolation stress [35,36].

### 3.2. Potential Pathomechanisms of Cardiovascular and Behavioral Alterations in SHR

Recently, it was shown in our laboratory that SHRs represent a low-to-normal renin and low-to-normal angiotensin model of hypertension, as levels of renin, Ang II, and other components of the RAAS (including angiotensin III, angiotensin IV, and angiotensin 1–7) were found to be normal or decreased rather than elevated [37]. Thus, RAAS does not seem to play a substantial role in the investigated parameters of SHRs.

However, the fact that HR, an indicator of adrenergic system activity, is significantly elevated in SHRs [37], and that in previous studies, renal sympathetic denervation prevented the development of hypertension and left ventricular remodeling in SHRs [38], suggests that the hemodynamic and structural alterations observed in SHRs may be primarily driven by activation of the sympathetic nervous system (SNS).

The presumption that activation of the adrenergic system in SHRs plays a crucial role in this rat strain is, to some extent, consistent with our findings of anxiolytic-like behavior. Specifically, SHRs exhibited excessive courage by entering illuminated or unprotected areas compared to the Wistar control rats. The previous literature has repeatedly documented spontaneous hyperactivity and hyperactivity to novel stimuli in SHRs [39,40]. Through selective inbreeding between SHRs and normotensive Wistar rats, two models were developed that separated hyperactivity from hypertension. The so-called Wistar-Kyoto hyperactive (WKHA) rat strain shows hyperactivity without accompanying hypertension, while the Wistar-Kyoto hypertensive (WKHT) rat strain is hypertensive but does not display hyperactivity [41]. Such a separation of pathological behavior from the influence of hypertension allows for a more targeted study of hemodynamic and psychogenic alterations [39]. The SHR model, which is frequently used in cardiovascular research and exhibits spontaneous and stress-induced hyperactivity, has also been used as a model for studying attention deficit hyperactivity disorder [27,42]. Interestingly, the exaggerated sympathetic adrenal medullary reaction to acute stress, demonstrated by increased catecholamine release, has been observed in SHRs and WKHA but not in the WKHT strain [43], suggesting that activation of the SNS may be linked to the behavioral pattern in SHRs but not to their elevated BP. These findings raise the consideration about a potential relationship between behavioral patterns in SHRs and other basic cardiovascular parameters related to BP, such as HR, LVW, or LVHP.

### 3.3. The Correlation Analysis of Anxiety-like Behavior and Cardiovascular Parameters in Untreated SHRs

The correlation analysis of the results of individual tests of anxiety-like behavior (EPM, OF, and LDB) and SBP, HR, and relative LVW, and LVHP did not show any evident relationship between anxiety level and the mentioned cardiovascular variables. This allows the assumption that the anxiety level in SHRs is independent of the accompanying hypertension, increased HR, and structural alterations, such as left ventricular hypertrophy and fibrosis. Rather, it seems likely that the changes in behavior in the sense of anxiolysis—i.e., “bolder” behavior—are an inherent, and are probably a genetically determined property in SHRs. However, similar results of correlation analysis were achieved not only for untreated SHRs but also for the Wistar control rats and for all treated groups. Thus, it seems that the level of anxiety, like behavior, does not correlate with cardiovascular parameters such as SBP, HR, LVW, and LVHP. The absence of correlation may be model-specific, and it cannot be ruled out that in different rat models the correlation between hemodynamic and behavioral phenotypes could be positive. However, the number of rats used in each group (n = 13) represents a sufficient cohort for reliable statistical power to support the belief that the absence of correlation may be real and not just a calculated bias. 

### 3.4. The Effects of ARNI on Cardiovascular and Behavioral Phenotype of SHRs

The chronic administration of ARNI had no significant effect on anxiety-like manifestations in SHRs. It seems that this finding may appear contradictory to clinical observations, where ARNI has demonstrated anxiolytic effects. Specifically, switching from enalapril to ARNI in symptomatic patients with heart failure not only improved cardiovascular outcomes but was also associated with reductions in depressive and anxiety symptoms [27,28]. Moreover, another study reported that the anxiolytic effect of ARNI could emerge as early as one week after the initiation of therapy [29]. However, it is important to note that in our study, SHRs did not exhibit anxiety-like behavior; rather, they displayed an opposite phenotype characterized by anxiolytic or bold behavior. Therefore, the anxiolytic potential of ARNI, obviously observed in patients with clinically relevant anxiety or depression, had no pathological behavioral substrate upon which to act in this experimental model. On the other hand, the fact that ARNI treatment in SHRs, despite significantly reducing hypertension and cardiac remodeling in this study, and fully reversing both systolic and diastolic dysfunction in our previous work [37], did not alter behavioral patterns, further supports the conclusion drawn from the correlation analysis: that the behavioral phenotype of SHRs does not associate with hemodynamic status and structural cardiac changes and is more likely determined by inherent neurobehavioral traits of the SHR strain.

### 3.5. The Effects of Captopril and Ivabradine on Cardiovascular and Behavioral Phenotype of SHRs

Similar to ARNI, which reduced SBP, HR, and relative LVW significantly and LVHP numerically, ivabradine and captopril also exerted hemodynamic and structural heart protection in this experiment. These findings are consistent with our previous studies using ARNI or captopril in L-NAME-induced hypertension [44], continuous light, or lactacystine-induced pre-hypertension [45,46] or SHRs [37,47], where ARNI or captopril revealed cardiovascular protection and improved LV systolic and diastolic function [37,45]. Analogically, ivabradine prevented cardiovascular alterations in L-NAME-hypertension [37,48] and in isoproterenol-induced heart damage [49]. The effects of these drugs on anxiety levels are not consistent. In experimental conditions, captopril exerted an anxiolytic–like effect in doxorubicin-treated rats in a preventive study [20]. In patients with anxiety or panic with stress-induced excessive hypertension, sublingual captopril and diazepam similarly reduced blood pressure [21], and captopril and enalapril improved depressed mood in hypertensive patients [22]. On the other hand, chronic high doses of captopril induced depression-like symptoms in mice via Treg cell reduction [50]. Interestingly, ivabradine showed a neutral effect on anxiety in L-NAME hypertension [51], but it reduced stress-related anxiety in patients with angina pectoris and heart failure, likely due to its heart rate-reducing effect [52]. In the current experiment, neither captopril nor ivabradine significantly modified the anxiolytic behavior in SHRs. Moreover, the correlation analysis showed no consistent relationship between cardiovascular parameters of hypertension, such as SBP, HR, LV, or LVHP, and behavioral outcomes in EPM, OF, and LDB. This suggests that the behavioral patterns seen in the group studied were independent of cardiovascular parameters, even under chronic treatment with cardiovascular protectives.

### 3.6. Conclusions

SHRs exhibited a reduced level of anxiety-like behavior—an anxiolytic/bold behavior pattern in several established anxiety assessment tests, along with elevation of SBP, HR, LVW, and LVHP. Chronic administration of ARNI, captopril, or ivabradine reduced SBP and HR and attenuated cardiac remodeling in SHRs. However, these treatments did not affect the anxiety-like behavior profile in SHRs. Correlation analysis further demonstrated that the level of anxiety-like behavior in SHRs and the other investigated group was not related to fundamental hemodynamic parameters or structural remodeling of the left ventricle. These findings suggest that anxiety-like behavior represents an intrinsic trait of both SHRs and Wistar rats, independent of cardiovascular alterations.

## 4. Materials and Methods

### 4.1. Animals and Treatment

**Figure 10:**
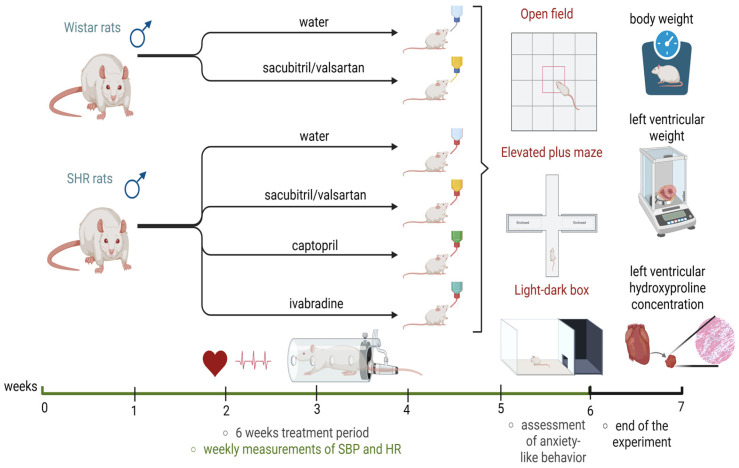
Experimental timeline (created in https://BioRender.com). SHRs—spontaneously hypertensive rats; SBP—systolic blood pressure; HR—heart rate.

The present study used twelve-week-old male Wistar albino and spontaneously hypertensive rats (Department of Toxicology and Laboratory Animals Breeding, Slovak Academy of Science, Dobra Voda, Slovakia). Males were individually housed in polycarbonate cages (41 cm × 28 cm × 15 cm) and maintained in a temperature- (22 ± 2 °C) and humidity-controlled (55 ± 10%) room with a 12:12 h light/dark schedule. The rats had access to standard food and water ad libitum. Male rats were randomly divided into six groups (n = 13 per group) and treated for six weeks as follows: control group:

- C group—Wistar rats with no treatment;
- C + ARNI group—Wistar rats treated with ARNI (68 mg/kg/day; Novartis, Basel, Switzerland);
- SHR group—SHRs with no treatment;
- SHR + ARNI group—SHRs treated with ARNI (68 mg/kg/day);
- SHR + IVA group—SHRs treated with ivabradine (10 mg/kg/day; Servier, Suresnes, France);
- SHR + CAP group—SHRs treated with captopril (100 mg/kg/day; Cayman Chemical Company, Ann Arbor, MI, USA; Figure 10).

The medications were administered via drinking water, with concentrations adjusted according to daily water intake (Figure 10). All experimental procedures were approved by the Ethics Committee of the Institute of Pathophysiology, Faculty of Medicine, Comenius University, Bratislava, Slovakia (approval number: 809/19-221/3; approval date: 23 April 2019) and were conducted according to European Union (EU) Directive 2010/63/EU and Slovak legislation.

### 4.2. Assessments of Hemodynamic Function, Behavioral Testing, and Cardiac Structural Parameters

SBP and HR were measured using a non-invasive tail-cuff plethysmograph (Hugo-Sachs Elektronik, Freiburg, Germany), with baseline measurements recorded before treatment and weekly until week six (Figure 10). Each measurement was performed three times per session, and the reported values represented the average of these three readings.

In the sixth week of treatment, rats underwent a battery of behavioral tests to assess anxiety-like behavior (Figure 10).

The OF test was conducted in a square arena (100 × 100 cm) divided into central and border zones. Rats were placed in the center of the arena, which was slightly illuminated with white light, and allowed to explore it freely for five minutes. Anxiolytic-like behavior was assessed based on the entries and amount of time spent in the central zone of the OF arena. The time spent in the central zone was expressed as a percentage of the total observation time [53,54].

The EPM apparatus consisted of four arms: two open (without walls, 45 × 10 cm), slightly illuminated with white light, and two closed arms (45 × 10 × 40 cm) extending from a central platform (10 × 10 cm). Each arm of the apparatus was attached to metal legs, elevating the maze to a height of 50 cm above the floor. Rats were placed at the end of the open arm, facing away from the maze, and were allowed to freely explore the maze for five minutes. The time spent on the open arms (the percentage of the total observation time) and the number of open arm entries were considered to be indicators of behavior [53,54].

The LDB test was performed in a rectangular box (40 × 55 cm) divided into two compartments: a brightly lit compartment and a dark compartment (covered by a lid), connected by an opening. Rats were placed in the light compartment and allowed to explore the box freely for five minutes. In addition to the frequency of entries into the light compartment, anxiolytic-like behavior was calculated as the relative time-percentage of the total time spent in the light compartment of the LDB [53].

The arena of all behavioral mazes was cleaned with Incidur spray (Ecolab, Dusseldorf, Germany) between each trial. Animal behavior was recorded and analyzed using the Noldus EthoVision XT 16 video tracking system (Noldus Information Technology, Wageningen, The Netherlands), using a camera mounted above the mazes. The center point detection of the animal body was utilized for evaluation of animal behavior in the arena of the OF, EPM, and LDB, which was automatically analyzed by the EthoVision software.

At the end of the six-week treatment period, rats were euthanized by isoflurane (Vetpharma Animal Health, S.L, Barcelona, Spain) inhalation (5% isoflurane, 95% oxygen). Body weight and LVW were measured, and LVW ratios were calculated as an indicator of left ventricular hypertrophy. Left ventricular tissue samples, collected and stored at −80 °C, were subsequently used for the assessment of LVHP, expressed in milligrams per total left ventricular weight. LVHP was measured as described by [55,56] and served as a marker of left ventricular fibrosis (Figure 10).

### 4.3. Statistical Analysis

Statistical analyses were performed using IBM SPSS Statistics for Windows, version 31.0.1.0 (49) (IBM Corp., Armonk, NY, USA). A one-way ANOVA was used to evaluate SBP, HR, LVW, LVHP, and the behavioral test results, and the corresponding *F* statistics with degrees of freedom and *p*-values were reported. For Bonferroni post hoc pairwise comparisons, *t*-statistics, degrees of freedom, and adjusted *p*-value are reported. Differences were considered statistically significant at *p* < 0.05. Results are reported as mean ± standard error mean (SEM). Data normality was assessed using the Shapiro–Wilk test for each group. Spearman’s correlation analysis was used to assess the association between cardiovascular parameters (SBP, HR, LVW, and LVHP) and anxiety-like behavioral parameters. For each correlation, Spearman’s rho, the corresponding *p*-value, and the 95% confidence interval (CI) were reported. Data visualizations were created using Python (version 3.11.9; Python Software Foundation, Beaverton, OR, USA) with the pandas (version 2.2.3) and matplotlib (version 3.10.0) libraries, in Visual Studio Code (Microsoft Corporation, Redmond, WA, USA).

## 5. Limitations

Considering that SHRs exhibit anxiolytic behavior associated with impulsivity and enhanced motor activity [57], the question arises, which molecular signaling system underlies this complex phenotypic behavioral trait? Data suggest that behavioral alterations of SHRs may be associated with a dopamine transfer deficit [58,59,60] and hypothalamic–pituitary–adrenal system dysfunction [61]. It would be valuable to investigate the effect of ARNI on behavioral manifestations in relation to dopaminergic or stress pathways and to describe the correlations of these humoral alterations with cardiovascular parameters of SHRs and ARNI-treated rats. It should also be considered that the deleterious ACE-AngII-AT1R system [62] or protective ACE2-Ang1-7-MAS [63] pathways in the brain structures [62,63], as well as potential modulation of the hematoencephalic barrier, may be modified in SHRs and under the treatment with ARNI. The disclosure of these molecular signal potential alterations may contribute to better targeting anxiolytic and cardiovascular therapy in experimental conditions and clinical settings.

Our animal experiments, like most previous works with SHRs and Wistar rats, were performed exclusively on males. A number of literature data report on sex differences in SHRs regarding hypertension development [64,65] or anxiety and depression manifestations [66,67], which could be reflected in a sex-determined variations in response to therapeutic intervention. Therefore, a comparison of the effects of ARNI and other cardiovascular agents on behavior in male and female groups could be of considerable interest and could help to eliminate sex as a confounding factor in the research of drug effects on cardiovascular and behavioral pathology. However, such investigations exceeded our laboratory possibilities and are a challenge for future experiments.

## Data Availability

The original contributions presented in this study are included in the article. Further inquiries can be directed to the corresponding author.

## Abbreviations

The following abbreviations are used in this manuscript:

ACE	Angiotensin-converting enzyme
Ang II	Angiotensin II
ANP	Atrial natriuretic peptides
ARNI	Sacubitril/valsartan
AT1R	Angiotensin II type 1 receptor
BNP	Brain natriuretic peptides
C	Wistar control rats
CAP	Captopril
CI	Confidence interval
CVD	Cardiovascular disease
EPM	Elevated plus maze test
HR	Heart rate
If	Hyperpolarization-activated cyclic nucleotide-gated ‘funny’ channel
IVA	Ivabradine
LDB	Light–dark box test
L-NAME	Nω-nitro-L-arginine methyl ester
LVHP	Left ventricular hydroxyproline concentration
LVW	Left ventricular weight
OF	Open field test
RAAS	Renin–angiotensin–aldosterone system
SBP	Systolic blood pressure
SEM	Standard error mean
SHR	Spontaneously hypertensive rats
SNS	Sympathetic nervous system
WKHA	Wistar-Kyoto hyperactive rats
WKHT	Wistar-Kyoto hypertensive rats
WKY	Wistar-Kyoto rat

## Appendix A

**Table A1:** Correlation between cardiovascular parameters and anxiety-like behavioral parameters in the C group.

CVS	Behavioral Parameter	Spearman’s Rho	*p*-Value	95% CI	
SBP	OF, central zone entries	0.19	0.54	−0.42	0.68
SBP	OF, time spent in central zone	0.21	0.49	−0.40	0.69
SBP	EPM, open-arm entries	−0.07	0.82	−0.61	0.51
SBP	EPM, time spent on open arms	−0.13	0.67	−0.65	0.47
SBP	LDB, light box entries	0.07	0.81	−0.51	0.61
SBP	LDB, time spent in light zone	0.08	0.79	−0.51	0.62
HR	OF, central zone entries	0.20	0.51	−0.41	0.69
HR	OF, time spent in central zone	0.09	0.76	−0.50	0.62
HR	EPM, open-arm entries	−0.15	0.62	−0.66	0.45
HR	EPM, time spent on open arms	−0.20	0.51	−0.69	0.41
HR	LDB, light box entries	0.10	0.74	−0.49	0.63
HR	LDB, time spent in light zone	−0.23	0.46	−0.70	0.39
LVW	OF, central zone entries	−0.25	0.40	−0.72	0.36
LVW	OF, time spent in central zone	−0.27	0.37	−0.72	0.35
LVW	EPM, open-arm entries	−0.62	0.02 *	−0.88	−0.09
LVW	EPM, time spent on open arms	−0.57	0.04 *	−0.86	−0.01
LVW	LDB, light box entries	0.06	0.84	−0.52	0.61
LVW	LDB, time spent in light zone	0.01	0.99	−0.56	0.57
LVHP	OF, central zone entries	−0.39	0.19	−0.78	0.23
LVHP	OF, time spent in central zone	−0.31	0.30	−0.75	0.30
LVHP	EPM, open-arm entries	0.15	0.63	−0.45	0.66
LVHP	EPM, time spent on open arms	0.21	0.50	−0.41	0.69
LVHP	LDB, light box entries	0.54	0.06	−0.03	0.85
LVHP	LDB, time spent in light zone	0.43	0.14	−0.18	0.80

Nonparametric correlation analysis was used to evaluate the associations; Spearman’s rho with the corresponding *p*-value and confidence interval (CI 95%) are reported. * Significant at *p* < 0.05. Abbreviations: CVS—cardiovascular parameters; SBP—systolic blood pressure; HR—heart rate; LVW—left ventricular weight; LVHP—left ventricular hydroxyproline concentration; EPM—Elevated plus maze test; OF—Open field test; and LDB—Light–dark box test.

**Table A2:** Correlation between cardiovascular parameters and anxiety-like behavioral parameters in the C + ARNI group.

CVS	Behavioral Parameter	Spearman’s Rho	*p*-Value	95% CI	
SBP	OF, central zone entries	−0.19	0.56	−0.68	0.43
SBP	OF, time spent in central zone	0.13	0.67	−0.47	0.65
SBP	EPM, open-arm entries	0.41	0.17	−0.20	0.79
SBP	EPM, time spent on open arms	0.20	0.51	−0.41	0.69
SBP	LDB, light box entries	−0.19	0.53	−0.68	0.42
SBP	LDB, time spent in light zone	0.30	0.33	−0.32	0.74
HR	OF, central zone entries	−0.19	0.54	−0.68	0.42
HR	OF, time spent in central zone	−0.48	0.10	−0.82	0.12
HR	EPM, open-arm entries	−0.42	0.15	−0.80	0.19
HR	EPM, time spent on open arms	−0.08	0.80	−0.61	0.51
HR	LDB, light box entries	0.17	0.59	−0.44	0.67
HR	LDB, time spent in light zone	0.17	0.58	−0.44	0.67
LVW	OF, central zone entries	0.02	0.95	−0.55	0.58
LVW	OF, time spent in central zone	−0.33	0.28	−0.75	0.29
LVW	EPM, open-arm entries	−0.45	0.12	−0.81	0.15
LVW	EPM, time spent on open arms	−0.01	0.97	−0.57	0.56
LVW	LDB, light box entries	0.02	0.95	−0.55	0.58
LVW	LDB, time spent in light zone	−0.28	0.35	−0.73	0.33
LVHP	OF, central zone entries	−0.26	0.40	−0.72	0.36
LVHP	OF, time spent in central zone	−0.58	0.04 *	−0.86	−0.03
LVHP	EPM, open-arm entries	−0.49	0.09	−0.83	0.10
LVHP	EPM, time spent on open arms	−0.24	0.43	−0.71	0.37
LVHP	LDB, light box entries	−0.01	0.98	−0.57	0.56
LVHP	LDB, time spent in light zone	−0.32	0.29	−0.75	0.30

Nonparametric correlation analysis was used to evaluate the associations; Spearman’s rho with the corresponding *p*-value and confidence interval (CI 95%) are reported. * Significant at *p* < 0.05. Abbreviations: CVS—cardiovascular parameters; SBP—systolic blood pressure; HR—heart rate; LVW—left ventricular weight; LVHP—left ventricular hydroxyproline concentration; EPM—Elevated plus maze test; OF—Open field test; and LDB—Light–dark box test.

**Table A3:** Correlation between cardiovascular parameters and anxiety-like behavioral parameters in the SHR group.

CVS	Behavioral Parameter	Spearman’s Rho	*p*-Value	95% CI	
SBP	OF, central zone entries	−0.11	0.73	−0.63	0.49
SBP	OF, time spent in central zone	−0.30	0.32	−0.74	0.32
SBP	EPM, open-arm entries	−0.69	0.009 **	−0.90	−0.21
SBP	EPM, time spent on open arms	−0.32	0.28	−0.75	0.30
SBP	LDB, light box entries	−0.16	0.61	−0.66	0.45
SBP	LDB, time spent in light zone	−0.14	0.66	−0.65	0.46
HR	OF, central zone entries	−0.09	0.77	−0.62	0.50
HR	OF, time spent in central zone	0.10	0.75	−0.49	0.63
HR	EPM, open-arm entries	0.02	0.94	−0.55	0.58
HR	EPM, time spent on open arms	−0.23	0.45	−0.70	0.39
HR	LDB, light box entries	−0.01	0.97	−0.57	0.56
HR	LDB, time spent in light zone	0.60	0.03 *	−0.06	0.87
LVW	OF, central zone entries	−0.31	0.30	−0.74	0.31
LVW	OF, time spent in central zone	−0.43	0.14	−0.80	0.18
LVW	EPM, open-arm entries	0.36	0.23	−0.26	0.77
LVW	EPM, time spent on open arms	0.59	0.03 *	0.04	0.87
LVW	LDB, light box entries	0.44	0.14	−0.17	0.80
LVW	LDB, time spent in light zone	−0.48	0.09	−0.82	0.11
LVHP	OF, central zone entries	0.68	0.01 *	0.18	0.90
LVHP	OF, time spent in central zone	0.63	0.02 *	0.11	0.88
LVHP	EPM, open-arm entries	0.02	0.95	−0.55	0.58
LVHP	EPM, time spent on open arms	−0.20	0.52	−0.68	0.41
LVHP	LDB, light box entries	−0.15	0.64	−0.66	0.46
LVHP	LDB, time spent in light zone	0.28	0.36	−0.34	0.73

Nonparametric correlation analysis was used to evaluate the associations; Spearman’s rho with the corresponding *p*-value and confidence interval (CI 95%) are reported. * Significant at *p* < 0.05; ** Significant at *p* < 0.01. Abbreviations: CVS—cardiovascular parameters; SBP—systolic blood pressure; HR—heart rate; LVW—left ventricular weight; LVHP—left ventricular hydroxyproline concentration; EPM—Elevated plus maze test; OF—Open field test; and LDB—Light–Dark box test.

**Table A4:** Correlation between cardiovascular parameters and anxiety-like behavioral parameters in the SHR + ARNI group.

CVS	Behavioral Parameter	Spearman’s Rho	*p*-Value	95% CI	
SBP	OF, central zone entries	0.09	0.77	−0.50	0.62
SBP	OF, time spent in central zone	0.10	0.75	−0.49	0.63
SBP	EPM, open-arm entries	0.45	0.12	−0.15	0.81
SBP	EPM, time spent on open arms	0.71	0.006 **	0.25	0.91
SBP	LDB, light box entries	0.16	0.61	−0.45	0.66
SBP	LDB, time spent in light zone	0.19	0.54	−0.42	0.68
HR	OF, central zone entries	0.15	0.63	−0.45	0.66
HR	OF, time spent in central zone	−0.10	0.75	−0.63	0.49
HR	EPM, open-arm entries	−0.34	0.25	−0.76	0.28
HR	EPM, time spent on open arms	−0.45	0.12	−0.81	0.15
HR	LDB, light box entries	−0.13	0.67	−0.65	0.47
HR	LDB, time spent in light zone	−0.02	0.94	−0.58	0.55
LVW	OF, central zone entries	−0.08	0.79	−0.62	0.51
LVW	OF, time spent in central zone	−0.09	0.77	−0.62	0.50
LVW	EPM, open-arm entries	0.17	0.59	−0.44	0.67
LVW	EPM, time spent on open arms	0.33	0.27	−0.29	0.75
LVW	LDB, light box entries	−0.04	0.91	−0.59	0.54
LVW	LDB, time spent in light zone	0.04	0.90	−0.54	0.59
LVHP	OF, central zone entries	0.27	0.38	−0.35	0.72
LVHP	OF, time spent in central zone	0.22	0.48	−0.40	0.70
LVHP	EPM, open-arm entries	−0.24	0.44	−0.71	0.38
LVHP	EPM, time spent on open arms	−0.46	0.11	−0.81	0.14
LVHP	LDB, light box entries	−0.51	0.08	−0.83	0.08
LVHP	LDB, time spent in light zone	0.50	0.08	−0.09	0.83

Nonparametric correlation analysis was used to evaluate the associations; Spearman’s rho with the corresponding *p*-value and confidence interval (CI 95%) are reported. ** Significant at *p* < 0.01. Abbreviations: CVS—cardiovascular parameters; SBP—systolic blood pressure; HR—heart rate; LVW—left ventricular weight; LVHP—left ventricular hydroxyproline concentration; EPM—Elevated plus maze test; OF—Open field test; and LDB—Light–dark box test.

**Table A5:** Correlation between cardiovascular parameters and anxiety-like behavioral parameters in the SHR + CAP group.

CVS	Behavioral Parameter	Spearman’s Rho	*p*-Value	95% CI	
SBP	OF, central zone entries	0.17	0.58	−0.43	0.67
SBP	OF, time spent in central zone	0.45	0.12	−0.15	0.81
SBP	EPM, open-arm entries	0.36	0.23	−0.26	0.77
SBP	EPM, time spent on open arms	0.30	0.32	−0.32	0.74
SBP	LDB, light box entries	−0.50	0.09	−0.83	0.10
SBP	LDB, time spent in light zone	−0.23	0.46	−0.70	0.39
HR	OF, central zone entries	−0.09	0.76	−0.62	0.50
HR	OF, time spent in central zone	0.21	0.49	−0.40	0.69
HR	EPM, open-arm entries	0.19	0.54	−0.42	0.68
HR	EPM, time spent on open arms	0.29	0.33	−0.32	0.74
HR	LDB, light box entries	0.09	0.77	−0.50	0.62
HR	LDB, time spent in light zone	−0.01	0.97	−0.57	0.56
LVW	OF, central zone entries	0.40	0.17	−0.21	0.79
LVW	OF, time spent in central zone	0.25	0.40	−0.36	0.72
LVW	EPM, open-arm entries	−0.05	0.88	−0.60	0.53
LVW	EPM, time spent on open arms	−0.22	0.47	−0.70	0.39
LVW	LDB, light box entries	0.39	0.19	−0.22	0.78
LVW	LDB, time spent in light zone	0.01	0.97	−0.56	0.57
LVHP	OF, central zone entries	−0.62	0.02 *	−0.88	−0.09
LVHP	OF, time spent in central zone	−0.38	0.20	−0.78	0.23
LVHP	EPM, open-arm entries	0.11	0.71	−0.48	0.64
LVHP	EPM, time spent on open arms	0.04	0.89	−0.53	0.59
LVHP	LDB, light box entries	0.30	0.32	−0.32	0.74
LVHP	LDB, time spent in light zone	0.03	0.92	−0.54	0.59

Nonparametric correlation analysis was used to evaluate the associations; Spearman’s rho with the corresponding *p*-value and confidence interval (CI 95%) are reported. * Significant at *p* < 0.05. Abbreviations: CVS—cardiovascular parameters; SBP—systolic blood pressure; HR—heart rate; LVW—left ventricular weight; LVHP—left ventricular hydroxyproline concentration; EPM—Elevated plus maze test; OF—Open field test; and LDB—Light–dark box.

**Table A6:** Correlation between cardiovascular parameters and anxiety-like behavioral parameters in the SHR + IVA group.

CVS	Behavioral Parameter	Spearman’s Rho	*p*-Value	95% CI	
SBP	OF, central zone entries	−0.37	0.22	−0.77	0.25
SBP	OF, time spent in central zone	0.51	0.07	−0.07	0.83
SBP	EPM, open-arm entries	−0.25	0.42	−0.71	0.37
SBP	EPM, time spent on open arms	−0.03	0.92	−0.58	0.54
SBP	LDB, light box entries	0.06	0.84	−0.52	0.60
SBP	LDB, time spent in light zone	−0.23	0.44	−0.71	0.38
HR	OF, central zone entries	−0.02	0.95	−0.58	0.55
HR	OF, time spent in central zone	0.23	0.46	−0.39	0.70
HR	EPM, open-arm entries	0.27	0.37	−0.35	0.72
HR	EPM, time spent on open arms	0.55	0.05	−0.02	0.85
HR	LDB, light box entries	−0.29	0.34	−0.73	0.33
HR	LDB, time spent in light zone	0.31	0.30	−0.30	0.75
LVW	OF, central zone entries	−0.02	0.94	−0.58	0.55
LVW	OF, time spent in central zone	−0.09	0.77	−0.62	0.50
LVW	EPM, open-arm entries	−0.04	0.89	−0.59	0.53
LVW	EPM, time spent on open arms	0.23	0.44	−0.38	0.71
LVW	LDB, light box entries	−0.10	0.74	−0.63	0.49
LVW	LDB, time spent in light zone	0.36	0.23	−0.26	0.77
LVHP	OF, central zone entries	0.16	0.61	−0.45	0.66
LVHP	OF, time spent in central zone	0.49	0.09	−0.11	0.82
LVHP	EPM, open-arm entries	−0.27	0.37	−0.72	0.35
LVHP	EPM, time spent on open arms	−0.14	0.65	−0.65	0.46
LVHP	LDB, light box entries	0.67	0.01 *	0.17	0.90
LVHP	LDB, time spent in light zone	0.12	0.71	−0.48	0.64

Nonparametric correlation analysis was used to evaluate the associations; Spearman’s rho with the corresponding *p*-value and confidence interval (CI 95%) are reported. * Significant at *p* < 0.05. Abbreviations: CVS—cardiovascular parameters; SBP—systolic blood pressure; HR—heart rate; LVW—left ventricular weight; LVHP—left ventricular hydroxyproline concentration; EPM—Elevated plus maze test; OF—Open field test; and LDB—Light–dark box test.
